# Agent-based modelling of iron cycling bacteria provides a framework for testing alternative environmental conditions and modes of action

**DOI:** 10.1098/rsos.211553

**Published:** 2022-05-18

**Authors:** Andre Then, Jan Ewald, Natalie Söllner, Rebecca E. Cooper, Kirsten Küsel, Bashar Ibrahim, Stefan Schuster

**Affiliations:** ^1^ Department of Bioinformatics, Matthias-Schleiden-Institute, University of Jena, Ernst-Abbe-Platz 2, 07743 Jena, Germany; ^2^ Institute of Biodiversity, Friedrich Schiller University Jena, Jena, Germany; ^3^ German Center for Integrative Biodiversity Research (iDiv) Halle-Jena-Leipzig, Leipzig, Germany; ^4^ Centre for Applied Mathematics and Bioinformatics, and Department of Mathematics and Natural Sciences, Gulf University for Science and Technology, Hawally 32093, Kuwait; ^5^ European Virus Bioinformatics Center, Leutragraben 1 07743 Jena, Germany

**Keywords:** agent-based modelling, bacterial interaction, *Shewanella*, *Sideroxydans*, modelling of mutualism, iron oxidation and reduction

## Abstract

Iron-reducing and iron-oxidizing bacteria are of interest in a variety of environmental and industrial applications. Such bacteria often co-occur at oxic-anoxic gradients in aquatic and terrestrial habitats. In this paper, we present the first computational agent-based model of microbial iron cycling, between the anaerobic ferric iron (Fe^3+^)-reducing bacteria *Shewanella* spp. and the microaerophilic ferrous iron (Fe^2+^)-oxidizing bacteria *Sideroxydans* spp. By including the key processes of reduction/oxidation, movement, adhesion, Fe^2+^-equilibration and nanoparticle formation, we derive a core model which enables hypothesis testing and prediction for different environmental conditions including temporal cycles of oxic and anoxic conditions. We compared (i) combinations of different Fe^3+^-reducing/Fe^2+^-oxidizing modes of action of the bacteria and (ii) system behaviour for different pH values. We predicted that the beneficial effect of a high number of iron-nanoparticles on the total Fe^3+^ reduction rate of the system is not only due to the faster reduction of these iron-nanoparticles, but also to the nanoparticles’ additional capacity to bind Fe^2+^ on their surfaces. Efficient iron-nanoparticle reduction is confined to pH around 6, being twice as high than at pH 7, whereas at pH 5 negligible reduction takes place. Furthermore, in accordance with experimental evidence our model showed that shorter oxic/anoxic periods exhibit a faster increase of total Fe^3+^ reduction rate than longer periods.

## 1. Introduction

Iron is the fourth most abundant element on the Earth's crust. It occurs mainly in two valence states: ferric iron (Fe^3+^) and ferrous iron (Fe^2+^). Microorganisms primarily control iron redox chemistry in most environments [1]. The respiration of iron, the microbial dissimilatory reduction of Fe^3+^ to Fe^2+^, is considered to be one of the earliest electron accepting processes on Earth [2], whereas molecular oxygen (O_2_), the preferred electron acceptor of many microorganisms and of higher organisms today, accumulated in the atmosphere and was much later released as waste product by phototrophic organisms. Today, respiration of Fe^3+^ is almost restricted to habitats where O_2_ is depleted like in anoxic sediments, gastrointestinal tracts, etc. Under anoxic conditions, insoluble iron oxyhydroxides are mainly reduced by Fe^3+^-reducing bacteria (FeRB). Besides contact-dependent reduction, FeRB are able to secrete electron shuttles, for example flavins, to indirectly reduce the insoluble iron oxyhydroxides [3]. Furthermore, chelating agents in the environment can increase the reduction rate via complexation of Fe^3+^ [4]. Their ubiquity and phylogenetic diversity makes this microbial metabolism globally significant [5]. In addition to the ability to use a solid phase mineral as an electron acceptor, FeRB such as *Shewanella* or *Geobacter* spp. can transfer electrons onto the surface of an electrode (anode), which led to the development of microbial fuel cells for the generation of electricity [6,7], or to other heavy metals and radionuclide contaminants which makes them useful in bioremediation [8–11].

The resulting Fe^2+^ can in turn be used by microorganisms as an electron donor for oxidation [12,13]. The re-oxidation of the biogenic Fe^2+^ to Fe^3+^ by iron-oxidizing bacteria (FeOB) can be coupled either to the reduction of O_2_ or nitrate. Under oxic conditions, microbial iron oxidation has to compete with the abiotic oxidation with O_2_, which is fast at circumneutral pH but negligible below pH 4. Furthermore, the reaction free energy of microbial iron oxidation is pH-dependent. The midpoint potential (*E*_*m*_) of O_2_/H_2_O increases from +0.82 V at pH 7 to +1.12 V at pH 2, making more energy available upon Fe^2+^-oxidation [14]. Thus, many FeOB prefer microoxic conditions and are called microaerophiles. Although the microbial Fe^2+^ oxidation coupled to the reduction of O_2_ has been known for more than a century, its geological significance had been discounted based on the rapid rate of abiotic Fe^2+^ oxidation. Only under very low pH conditions are some acidophilic bacteria able to execute both reduction and oxidation of iron [15].

Despite their thermodynamic and kinetic optima being bound to different geochemical niches, they have been shown to coexist in close proximity at aerobic–anaerobic interfaces [16]. Natural habitats also exhibit temporal oscillations of O_2_ concentrations, which are caused by fluctuating water levels, bioturbation and/or the release of O_2_ by plant roots during daytime. These conditions are characteristic for wetlands and sediments, which allow for FeRB and FeOB to be mutually active under anoxic or oxic phases at the same location during different times [17–19]. Those mixed communities of FeRB and FeOB have recently attracted the interest of both experimental and theoretical biology [20–22]. However, their interactions are hard to study in the laboratory because of their contrasting growth requirements (anoxic versus oxic conditions) and the susceptibility of abiotic Fe^2+^ oxidation at circumneutral pH [23,24]. These issues do not affect mathematical modelling. Thus, using modelling we can study the interaction of FeRB and FeOB within the same habitat by switching periodically between anoxic (Fe^3+^ reduction takes place) and oxic (Fe^2+^ oxidation takes place) conditions. By employing sensitivity analyses, we can further investigate how susceptible the total systems iron cycling is to different environmental parameters affecting both processes of iron cycling and the interaction between FeRB and FeOB. Although modelling cannot replace experimental studies, it can be very helpful in understanding the complex superposition of cooperation and competition in microbial consortia [25–28]. In the optimal case there is a fruitful interplay between mathematical modelling and experimental studies leading stepwise to an improved understanding of complex systems. In the following sections we will introduce some aspects and open questions related to FeRB/FeOB-communities where mathematical modelling could contribute to an improved understanding.

FeRB and FeOB have been shown to use various strategies with respect to electron transfer mechanisms, movement and with their ability to cope with abiotic reduction/oxidation processes [24]. However, it is still unclear how these different strategies affect the efficiency of iron cycling when both groups co-occur. For example, iron nanoparticles are faster reduced by FeRB and to a larger extent compared to bulk macroaggregates of the same iron phases with the same total surface area, due to surface passivation of the bulk macroaggregates with Fe^2+^ [29]. But as more nanoparticles can adhere to the FeRBs cell surface when it swims in the medium, there is a trade-off between achieving maximal bulk macroaggregate reduction upon adhesion and maximal nanoparticle catching and reduction upon locomotion. More nanoparticle sized iron oxyhydroxides are formed by Fe^2+^ oxidation through FeOB compared with abiotic reactions [20], which might explain why biogenic iron oxyhydroxides are preferred by FeRB. How and when iron nanoparticles formed by the FeOB are released into the liquid medium could affect the nanoparticle availability and consequently the reduction rate of the FeRB. We test hypotheses for different modes of action and assess how their interactions affect the iron cycling system.

We were particularly interested in investigating the influence of different adhesion and encrustation prevention modes of *Shewanella* spp. and *Sideroxydans* spp., respectively, on the efficiency of iron cycling. For both modes of action the mechanisms are not well understood while multiple hypotheses exist. The dominant forces for adhesion are electrostatic interactions which allow for quick attachment/detachment on mineral surfaces [30]. On the other hand, it has been observed that over time the adhesion of *Shewanella* spp. gets stronger and it is unlikely that those forces can be overcome by changes in electrostatic repulsion [31]. Previous studies also showed that adhesion is stronger under anoxic compared to oxic conditions [31]. This hints more toward an attachment mechanism associated with the comparatively slow synthesis and degradation of polymeric matrices.

FeOB must employ a mode of action to prevent cell encrustation by the rapid formation of insoluble oxyhydroxides in the presence of water after oxidation of soluble Fe^2+^ to Fe^3+^ to maintain an exchange of metabolites with the environment. Several options exist for FeOB to tackle this challenge [32]. For example, encrustation can be prevented by lowering the pH in close proximity to the cell surface [33], which lowers the tendency of Fe^3+^ to precipitate or Fe^3+^ already precipitated on the cell surface can be pushed off by the formation of membrane vesicles [34] or other unknown mechanisms leading to the shedding of surface encrusting iron nanoparticles piece by piece. As the mode of action employed by *Sideroxydans* spp. is so far unknown, we have tested the influence of these two different modes of action on the efficiency of iron cycling.

As outlined above, environmental parameters like pH affect abiotic reactions, especially Fe^2+^ oxidation, and the activity of the different groups and species of both FeRB and FeOB, which have different defined pH optima [1,35]. Furthermore, pH affects mineral aggregation [36]. By adjusting the parameters in our model, we can demonstrate how much each process (reduction/oxidation) of the iron cycling is affected under different pH conditions, and how pH will also affect the interaction between the FeRB and FeOB.

In this study, we develop a novel agent-based model (ABM) describing the aforementioned core processes of iron cycling mediated by a model FeRB and FeOB. We consider reduction/oxidation, movement, adhesion, iron nanoparticle growth and mineral dissolution/precipitation. ABMs are characterized by the rule-guided implementation of the behaviour of interacting units called ‘agents’, such as cells or organisms [25,37,38]. Those guiding rules often involve stochasticity, which leads to non-deterministic behaviour in contrast to the deterministic behaviour described by, for example, differential equation-based models. Most of the models involve spatiality as well, in the form of a two- or three-dimensional grid environment, in which agents interact with their neighbours, often leading to an emergent global behaviour. ABMs are regarded as particularly well suited to investigate emergent systems behaviour [39–41]. Therefore, in the context of microbiology, ABMs have been applied to phenomena like spatial organization of bacterial colonies [42,43], signal transduction in bacterial chemotaxis [26] and the explanation of immunological strategies and kinetics [44–46]. We preferred an ABM to other approaches like, for example, partial differential equation (PDE) models as ABM helps to integrate additional information that is not available otherwise. For example, capturing heterogeneous mixing and agent interactions enables it to give a more realistic representation of the system. Moreover, solving PDEs for a different set of parameters and optimization can get very complex since we likely have a stiff system. Finally, it would be difficult to describe particles of different sizes (nanoparticles versus bulk macroaggregates) by PDEs.

We deem the processes included in our model ubiquitous in systems where FeRB and FeOB co-occur. To provide a model, both general and flexible, we retrieved parameter values from published experimental studies not specific to just one model bacteria. The model is supposed to initiate the transition from the numerous experimental studies, which deal mostly with isolated aspects like measuring e.g. the reduction rate or the percentage of bacteria attached to minerals, to a systemic view of the FeRB/FeOB-interplay. We demonstrate the potential insights by examining the influence of different assumptions about core processes and environmental variables like pH on system variables such as for example the speed of iron reduction/oxidation. The resulting knowledge is of importance for the implementation of iron cycling bacteria for applied projects including bioremediation and electromicrobiology.

## 2. Methods and biological background

### 2.1. Modelling framework

**Figure 1.**
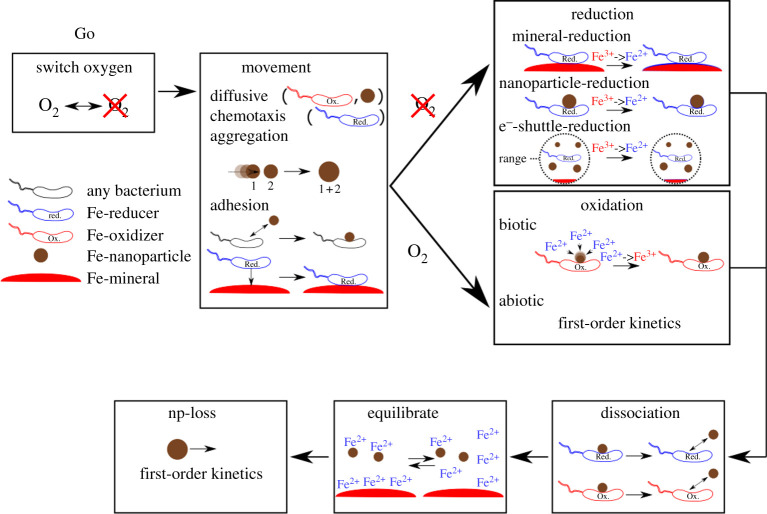
Schematic of the processes included in the proposed ABM. For further information on how the processes were implemented and which parameters were chosen, see Methods section.

We are using the free software NetLogo 3D 6.0.4 [39,47]. The environment of the ABM consists of a three-dimensional grid with dimensions of 21 × 21 × 21, resulting in a total of 9261 patches. In contrast to models consisting of ordinary differential equations, many of the processes include stochastic elements which results in a non-deterministic model. We implement FeRB, FeOB and nanoparticles as agents in our model. Patches can either be the medium bacteria traverse or bulk macroaggregates. Figure 1 gives an overview of the processes included in the model. The model switches between oxic and anoxic conditions. A csv-file specifies at which points of time the model switches between the two states. An ODD-protocol (Overview, Design concepts, Detail) of the model including a more detailed explanation of parameters and their estimation is found in the supplementary information electronic supplementary material, S1. The parameter values for all the runs are summarized in electronic supplementary material, Table S2. Where possible we based the parameter values on the model organisms *Shewanella* and *Sideroxydans* for FeRB and FeOB, respectively. In our model, we assume that the number of bacteria already reached an equilibrium and therefore does not change over time. Although this assumption may seem unrealistic, growth studies on *Shewanella* [48] and *Sideroxydans* [20] show that the doubling times of these model organisms for Fe^3+^-reduction/Fe^2+^-oxidation are in the range of multiple days. Our model only simulates two to three days and therefore we do not expect growth to have a dramatic influence on the model outcomes. In contrast to fast-growing bacteria like *E. coli*, which exhibit growth rates down to 20–30 min, we can reasonably expect the number of bacteria to stay within the same order of magnitude during the course of a simulation. To further justify this assumption, we conducted runs where microbial growth is included and found that only an expected linear increase in Fe^3+^-reduction and Fe^2+^-oxidation takes place (electronic supplementary material, S5).

Table 1 offers a short description for each parameter and states the process it is associated with. In the following sections, we will explain the parametrization and rules guiding the key processes of our model based on an enhanced version of the submodels sections of our ODD-protocol.

**Table 1.** Model parameters overview.

parameter name	description	associated process
fe-patch-percentage	percentage of patches which contain iron macroaggregate	world setup
fe-clustering	a parameter to control how densely the macroaggregate is clustered in the environment	world setup
initial-number-fe3reducer	number of FeRB at the beginning of the simulation	world setup
initial-number-fe2oxidizer	number of FeOB at the beginning of the simulation	world setup
initial-number-nanoparticle	number of nanoparticles at the beginning of the simulation	world setup
max-ticks	maximum number of ticks after which the situation is aborted	simulation control
speed-fe2-oxidizer	number of patches the FeOB traverses per tick	movement
speed-fe3-reducer	number of patches the FeRB traverses per tick	movement
iron-per-cubicnm	amount of iron in one cubicnm nanoparticle	nanoparticle characteristics
start-medium-fe2	Fe^2+^ at the beginning of the simulation	world setup
mineral-aggregation-probability	probability that two nanoparticles aggregate if they are on the same patch	aggregation
contact-reduction-rate	amount of Fe^3+^ reduced upon macroaggregate-adhesion per FeRB and tick	Fe^3+^-reduction
fe2-ads-tolerance	maximum amount of Fe^2+^ tolerated on the macroaggregate surface before detachment	macroaggregate adhesion
np-mineral-reduction-ratio	factor of improved reduction of nanoparticles compared to macroaggregate	nanoparticle reduction
abiotic-oxidation-ratio	share of total Fe^2+^ in the system which is reduced abiotically per tick	oxidation
np-attachment-threshold	minimum share of Fe^3+^ on total nanoparticle iron for the nanoparticle to adhere to the FeRB upon contact	nanoparticle adhesion
np-dissociation-threshold	maximum share of Fe^2+^ on total nanoparticle iron before the nanoparticle detaches from the FeRB	nanoparticle dissociation
shedding-diameter	diameter of the nanoparticle around which it is released from the cell surface of the FeOB	nanoparticle dissociation
fe2-equilibrium-rate	velocity with which Fe^2+^ assumes equilibrium between its different phases	equilibration
e-shuttle-range	range in patches around the FeRB within which it is able to reduce Fe^3+^ via electron shuttles	electron shuttle reduction
fe2-solid-ineq	share of Fe^2+^ which adheres to solid surfaces in equilibrium	equilibration
contact-oxidation-rate	The maximum amount of Fe^2+^ oxidized by a FeOB per tick	oxidation
np-loss-percentage	Percentage of nanoparticles leaving the system per tick	simulation control

### 2.2. Movement

The movement submodel calls the nanoparticles not bound to any bacterium, the oxidizers, and the reducers in this order to manage their movement and adhesion.

Free-floating nanoparticles: the nanoparticles exhibit diffusive movement with speed declining based on their diameter: (max(50 − 0.5 × diameter, 1)). If the target patch is inaccessible to agents, which is the case for patches in the core of a bulk mineral, i.e. surrounded only by bulk mineral patches in their neighbourhood, the target patch is changed to the bulk iron mineral patch on the edge of the same mineral cluster closest to the nanoparticle. Now, within the function for diffusive movement, if the caller is a nanoparticle, the function aggregate-adhere is called. This function manages the adhesion and aggregation of agents. The nanoparticle which just moved now calls every other free-floating nanoparticle and reducer on the same patch in a random order. If the called agent is a free-floating nanoparticle and a random number between 0 and 1 is smaller than *mineral-aggregation-probability*/100, the nanoparticles will aggregate, combining their iron contents and forming a new nanoparticle with a diameter corresponding to the combined amount of iron. If the called agent is a reducer, and the Fe^3+^ to total iron ratio of the nanoparticle is above the *np-attachment-threshold*, the nanoparticle will adhere to the reducer.

FeOB: based on comparative genomics [49] and the formation of distinct growth bands along opposing O_2_/Fe^2+^ gradients [13], it is highly likely that *Sideroxydans* spp. possess the capability for chemotactic movement based on sensing one or both of these species. However, as our model operates at a time scale where at every tick a complete diffusion of Fe^2+^ occurs, an implementation of chemotaxis for the FeOB would not have had any effect on the outcome of the model. The oxidizers therefor exhibit diffusive movement as described for the nanoparticles. They move with the velocity of *speed-fe2oxidizer*. As their surface is near-neutral charged and shows a high degree of hydrophilicity to prevent encrustation [30], we assume oxidizers to neither adhere to bulk mineral nor nanoparticles (after they are released from the oxidizers cell surface).

FeRB: for the reducers, it is first checked if they adhere to a bulk mineral patch or not. If they do not adhere, they move via chemotaxis with the speed *speed-fe3reducer*. If they adhere and the adsorbed Fe^2+^ on the mineral patch (fe2-ads) is higher than *fe2-ads-tolerance* they are either immediately released from the patch (adhesion-strategy: electrostatic) or with a certain probability which decreases with the time they have already been adhered to this patch and the presence of other bacteria on the mineral cluster in close proximity (adhesion-strategy: biofilm). Otherwise they just stay adhered to the mineral patch.

The FeRB *Shewanella* spp. is challenged by finding its way towards insoluble electron acceptors, which does not provide a chemical gradient by itself. Electrokinetic behaviour has been observed nevertheless [50], although the underlying mechanism is not ultimately solved yet. The observation that electrokinetic behaviour is limited to a small percentage of the overall population at a time, has led to the conclusion that electrokinetic motility is activated upon contact with the mineral containing the electron acceptor and preserved for a certain amount of time until it is either prolonged by renewed contact with the mineral or expires after a certain amount of time [50]. Upon chemotactic movement it is first checked if they have a bulk-mineral patch as a neighbour. If so, then the chemotactic mode is switched on. In the chemotactic mode, the tumblings (which are random reorientations) are trippled and the speed is doubled. These numbers were determined by Harris *et al*. [51]. Apart from this adjustment, chemotactic movement works the same as diffusive movement already described for the nanoparticles. For the reducers, the function aggregate-adhere is also called. They can either bind nanoparticles they encounter or adhere to a bulk mineral patch. The binding of nanoparticles is identical to the process of nanoparticles adhering to reducers explained above. Adhesion on the mineral takes place if the reducer is located on a bulk mineral patch and fe2-ads is smaller than *fe2-ads-tolerance*.

#### 2.2.1. Parametrization

*mineral-aggregation-probability*: the kinetics of nanoparticle aggregation remain unclear. Bose *et al.* [52] showed for example that hematite nanoparticles tend to aggregate in solution if their diameter is either 11 or 99 nm, whereas their tendency to do so is much weaker if they have a diameter of 30 or 43 nm. We therefore just assume a *mineral-aggregation-probability* of 100%. For lower pH, we assume a drop in *mineral-aggregation-probability* as the nanoparticles get more positively charged and the electrostatics repulsion between them gets stronger accordingly. This decrease has been quantified by Baalousha [36].

*fe2-ads-tolerance*: the situation is similar for the tolerance of Fe^2+^ adsorbed on bulk mineral for the reducer. At some point, the electrostatic repulsion and the accumulation of an iron redox-state which is not useable for the reducer's respiration has to make it detach from the mineral. Though we cannot directly measure this parameter, Roberts *et al.* [31] have shown that at neutral pH around 20% of the bacteria adhere to the mineral. We fitted the parameter so that during the course of an experiment it causes approximately the same percentage of iron reducers to attach to the surface. We arrived at a value of 300.

*np-attachment-threshold*: the information here is also unreliable and was just guessed to be around 50%. However, the sensitivity analysis in our paper explores the relative importance of these unclear parameters.

### 2.3. Iron reduction

The reduction submodel is called under anoxic conditions and manages all the reduction processes of the reducer, which include bulk mineral reduction, nanoparticle reduction and non-contact-dependent reduction via electron-shuttles.

If the reducer is attached to a bulk mineral patch it adds an amount of *contact-reduction-rate* to the Fe^2+^ adsorbed on the mineral patch (fe2-ads). Then the remaining reduction capacity is set to four times the *contact-reduction-rate*. This is based on the results of Marsili *et al.* [53], who showed that around 80% of Fe^3+^-reduction mediated by *Shewanella* occurs via electron shuttles. In their study, they did not include nanoparticles. As nanoparticles bind to the membrane protein responsible for reduction, MtrC, which is also responsible for recharging the electron-shuttles, they draw from the same resource, i.e. our reduction-capacity variable. First all bound nanoparticles with any Fe^3+^ are reduced. Based on their diameter they occupy a certain share of MtrC on the cell surface. Bonneville *et al.* [54] showed that there are around 10 000 MtrC-proteins on the cell surface. We assume they are equally distributed, so that the amount of MtrC occupied by one nanoparticle is its squared diameter over the cell's surface area times 10 000. The amount of Fe^3+^ reduced is then equal to the number of occupied MtrC divided by the total of MtrC of the cell times the *np-mineral-reduction-ratio* times the *contact-reduction-rate*. The transferred electrons divided by the *np-mineral-reduction-ratio* is then substracted from the reduction-capacity pool. The remaining reduction capacity is then equally distributed on bulk mineral patches within *e-shuttle-range*.

#### 2.3.1. Parametrization

*contact-reduction-rate*: from the cell densities and reduction rate reported in Lies *et al.* [55], one can easily calculate the reduction rate per cell. It amounts to $5.0\times 10^{7}\;\frac{{\mathrm{Fe}}^{3+}}{\min\cdot \mathrm{cell}}$ for *Shewanella*, lactate and Fe^3+^-hydroxide. The study includes electron-shuttle reduction. Because Marsili *et al.* [53] showed that around 80% of Fe^3+^-reduction mediated by *Shewanella* occurs via electron shuttles, we have to divide by five to obtain the *contact-reduction-rate*. This amounts to $1\times 10^{7}\;\frac{\mathrm{atoms}}{\mathrm{cell}\cdot \min}$.

*np-mineral-reduction-rate*: according to Bosch *et al.* [29] the reduction ratio of macroparticles was 6 to 70 $\frac{\mathrm{pmol}}{\mathrm{cell}\cdot h}\;\mathrm{compared}\;\mathrm{to}\;\mathrm{up}\;\mathrm{to}\;1255\frac{\mathrm{pmol}}{\mathrm{cell}\cdot h}$ for nanoparticles depending on the particular mineral. This was determined for *Geobacter* though. Normalized by surface area the improvement in reduction rates is approximately two orders of magnitude. We therefore assume a value of 100.

*e-shuttle-range*: the e-shuttle-range heavily depends on environmental conditions and has to our knowledge not been experimentally determined for any case study yet. As there is plenty of evidence for non-contact dependent reduction and our model should cover this process, we went for an arbitrary range of 10 patch lengths, which is equivalent to 20 μm.

### 2.4. Biotic oxidation

During biotic oxidation the FeOB precipitates and grows new nanoparticles on its surface. There are two possible strategies considered. Either the oxidizer lets nanoparticles precipitate on its cell surface and they grow up to a certain size (shedding), or they precipitate in close proximity to the cell and are immediately free-floating (low pH). The oxidizer either oxidizes its equal share of the total amount of dissolved Fe^2+^ or an amount equal to *contact-oxidation-rate*, whichever value is lower. This variable is called fe2-used-from-medium.

Shedding: if the oxidizer has less than 40 nanoparticles on its cell surface, it grows as many nanoparticles as it needs to reach 40 nanoparticles. Because there is no better literature evidence available, we estimated from electron micrographs [56] that 40 nanoparticles close to the *Sideroxydans* cell can represent the correct order of magnitude. The amount of Fe^2+^ spend on each nanoparticle is fe2-used-from-medium/40. The spend Fe^2+^ is subtracted from fe2-used-from-medium and the remaining Fe^2+^ is spent on all the nanoparticles on the cell surface based on their share of the total surface area occupied by nanoparticles (so larger nanoparticles get more oxidized and therefore grow faster).

Low pH: if nanoparticles are present on none of the surrounding patches, all fe2-used-from-medium is consumed to form a new nanoparticle on the same patch as the oxidizer. In contrast to the nanoparticles produced with the shedding strategy, these nanoparticles are not bound to the oxidizer and can freely move from the moment of their formation. If nanoparticles exist on the surrounding patches, the fe2-used-from-medium is equally distributed among all these nanoparticles.

#### 2.4.1. Parametrization

*Contact-oxidation-rate*: Maisch *et al.* [57] measured a maximum biological oxidation rate of $3.6\times 10^{7}\;\frac{{\mathrm{Fe}}^{2+}}{\mathrm{cell}\;\cdot \;\;\min}$.

### 2.5. Abiotic oxidation

FeOB have to compete with abiotic Fe^2+^ oxidation which dominates under high oxygen concentrations and neutral pH. Therefore, neutrophilic FeOB thrive under moderate oxygen concentrations and are hence called microaerophiles. The primary reaction of Fe^2+^ oxidation is given by [58]

$$2{\mathrm{Fe}}^{2+}+1/2O_{2}+2H^{+}\to 2{\mathrm{Fe}}^{3+}+H_{2}O.$$

This shows how a low pH drives the reaction towards Fe^2+^-oxidation. However, at high pH ferrous ions become less available as they are hydrated into other speciations which are more susceptible to abiotic oxidation, for example, ferrous hydroxide [59]:

$${\mathrm{Fe}}^{2+}+2H_{2}O\to \mathrm{Fe}{\left(\mathrm{OH}\right)}_{2}+2H^{+}.$$

Based on this equation, abiotic iron oxidation follows the first-order kinetics in regard to the concentration of Fe^2+^ [60]. That means a constant share of the total iron depended on the *abiotic-oxidation-ratio* is oxidized at each tick as long as the other relevant concentrations (oxygen, protons) do not change. This share of iron gets subtracted from the dissolved Fe^2+^, the Fe^2+^ on the bulk mineral surface and the nanoparticle Fe^2+^.

#### 2.5.1. Parametrization

*abiotic-oxidation-ratio*: Druschel *et al.* [13] have measured that the contribution of biotic oxidation to the total iron oxidation (biotic + abiotic) under biologically relevant oxygen concentrations ranges from 20 up to 90%. We used this as a rough estimate to calibrate the value for this parameter. A value of 0.01 results in a contribution of biotic oxidation to total iron oxidation of 20%. This value we chose for near neutral pH. For lower pH, we have to assume lower values, where the biotic oxidation contributes up to around 70% (for an abiotic-oxidation-ratio of 0.001).

### 2.6. Dissociation

This submodel manages the dissociation of nanoparticles from bacteria.

FeRB: if the partner is a reducer, the nanoparticle dissociates once the Fe^2+^-content gets above the *np-dissociation-threshold*. Surface passivation of the mineral by adsorbed Fe^2+^ is regarded as an important factor for preventing complete reduction [61]. However, the exact kinetics are unclear, depending on the mineral species, and are influenced by the presence of organic matter [62]. Therefore, a simplified representation of surface passivation is included in the model.

FeOB: if the partner is an oxidizer the nanoparticle dissociates once a random-number thrown from a distribution with a mean of the nanoparticle diameter and a standard-deviation of 2 is bigger than the shedding-parameter. This element of stochasticity was added, because it is unrealistic to assume that every nanoparticle exactly dissociates at a size corresponding to the *shedding-diameter*.

#### 2.6.1. Parametrization

*np-dissociation-threshold*: as already mentioned earlier, at what point nanoparticles dissociate from the reducer has not yet been quantitatively studied. We therefore arbitrarily chose a value of 0.7 and check its influence in our sensitivity analysis.

*shedding-diameter*: Hädrich *et al.* [20] measured the average diameter of nanoparticles in an environmental characteristic for Sideroxydans to be 10 nm. Therefore, we selected this value for the *shedding-diameter*.

### 2.7. Equilibration

This submodel manages the distribution adjustments of Fe^2+^ between bulk mineral, solution and nanoparticles. First the equilibration between bulk mineral patches and solution takes place. The bulk mineral patches are selected in random order. The difference between the adhered Fe^2+^ and the equilibrium-share *fe2-solid-ineq* is calculated. Then a fraction *fe2-equilibrium-rate* of Fe^2+^ is transferred in the direction of the equilibrium (either patch −> solution or solution −> patch.). *fe2-equilibrium-rate* defines how fast Fe^2+^ equilibrates. Afterwards nanoparticles and solution are equilibrated according to the same principle.

#### 2.7.1. Parametrization

*fe2-equilibrium-rate*: Hyacinthe *et al.* [63] showed that complete equilibration takes place within 2.5 h. We therefore calculated our *fe2-equilibrium-rate* to the minimum value that ensures that less than 5% of the initial deviation from equilibrium remains after 2.5 h. The corresponding value is 0.02.

*fe2-solid-ineq*: from Fe2+-distributions recorded by Wilmoth *et al.* [42], we estimated a value of 0.9.

### 2.8. np-loss

A certain share of nanoparticles (*np-loss-percentage*) gets lost each tick to simulate processes such as sedimentation, incorporation into bulk mineral, etc.

#### 2.8.1. Parametrization

*np-loss-percentage*: there can be no general value for the np-loss-percentage due to the complex variations in different environments. We went for 0.2, although in experimental set-ups, which are closed systems most of the time, a lower value might be closer to reality. For the validation of our hypothesis explaining an experimental pattern, we set this value to zero.

### 2.9. Influence of different modes of action

For the adhesion modes of *Shewanella* spp. the following two options have been considered:

(a) *Electrostatic:* the most important factor for adhesion/detachment is the charge of the surface to which *Shewanella* is attached. The cell surface charge of *Shewanella* spp. is negative, in opposition to the positive charge of iron minerals/nanoparticles below pH 7.8 [36]. Therefore, upon reduction the surface charge of the mineral/nanoparticle gets more negative and once it reaches the charge of the cell surface, the detachment occurs immediately. Thus, for this mode of action the adhesion/detachment probabilities are dependent only on the ratio of Fe^3+^ to total Fe.
(b) *Biofilm:* we incorporated this mode of action in our model by assigning to the attached *Shewanella* a probability for detachment per tick which declines with the time spent adhered on this surface. For oxic conditions, the process is similar but the probability of detachment for a certain time spent on the surface is always higher than the corresponding probability under anoxic conditions.

We assume either continuous shedding of diffusible nanoparticles with a chance proportional to the size of the nanoparticle or prevention of precipitation by a lowered pH close to the cell membrane of *Sideroxydans*:

(a) *Shedding:* the first mode of action is based on the continuous growth of nanoparticles on the cell surface and their stochastic repulsion from the cell surface once they are close to a size of 10 nm in diameter. *Sideroxydans lithotrophicus* ES-1 produced nano-sized biogenic Fe(III)-oxyhydroxides (10 nm) resulting from Fe^2+^ oxidation, which resembled DOM-impacted (dissolved organic matter) nanoparticles found in the minerotrophic Schlöppnerbrunnen fen [20]. We used this information as our best guess to calibrate the shedding parameter in our model.
(b) *Low pH:* the second mode of action allows Fe^3+^ precipitation on the closest structure accessible as a nucleation site instead of the cell surface of *Sideroxydans*. If there is no precipitation site available, the nascent Fe^3+^ precipitates into a new nanoparticle. Therefore, the nanoparticles are instantaneously released into the medium upon formation.

### 2.10. Influence of changing pH-values

As the different bacteria capable of reducing or oxidizing Fe are able to thrive under different pH-values, we wanted to capture the key influences of pH on the processes included in our model to compare how the interplay between FeOB and FeRB changes. The three factors which are influenced by pH in our model are (i) the stability of Fe^2+^ against abiotic oxidation, (ii) bacterial adhesion on mineral surfaces or nanoparticles and (iii) the aggregation of nanoparticles. As the rate equation of Fe^2+^-hydration contains the square of the proton concentration in the denominator, sinking pH values exhibit a strong effect on the speciation and overall lower the reaction velocity of abiotic Fe^2+^-oxidation [64]. Moreover, that makes the mineral surface more positive, so that the strength of adhesion between the negatively charged cell surface of *Shewanella* spp. and the mineral surface increases.

Roberts *et al.* [31] showed a negative correlation between the percentage of bacteria attached onto ferrihydrite and pH. Increasing the pH from 5 to 6 shows a sharp decrease in ferrihydrite-attached bacteria from approximately 80% to 20%. For nanoparticles, the maximum aggregation takes place around its point of zero charge which equals pH 7.8 [36]. The aforementioned study showed that lowering the pH from 7.0 down to 5.0 leads to a sharp decrease in aggregation as the electrostatic repulsion rises. Table 2 summarizes the influence of pH on these key processes.

**Table 2.** Summary of the influence of pH on key processes included in the model.

pH	5	6	7
abiotic oxidation rate	low	medium	high
*Shewanella* adhesion	high	low	low
nanoparticle aggregation	very low	low	maximum

### 2.11. Sensitivity analysis

The sensitivity analysis was conducted with Python 3.6. The NetLogo 6.0.4 runs were controlled from Python with the pynetlogo package v. 0.3 [65] and parallelized with ipyparallel 6.3.0. For the sensitivity analysis the SALib extension was used [66]. Thirteen parameters have been tested with a total of 14 000 runs. The parameter borders within which parameter values are sampled following a uniform distribution were set to approximately ±20% of our estimate for the corresponding parameter. First, second and total Sobol-indices were calculated. Those parameters give an estimate of the strength of the parameter's influence without interaction, its combined influence without interactions plus the interactions with one parameter at the same time, and its total influence considering all possible combinations of interactions on the model outcome, respectively [67]. The Python code for the set-up of the model runs and the subsequent analysis can be found in electronic supplementary material, S3 and S4, respectively.

## 3. Results and discussion

### 3.1. Demonstration of hypothesis testing on an experimental study

**Figure 2.**
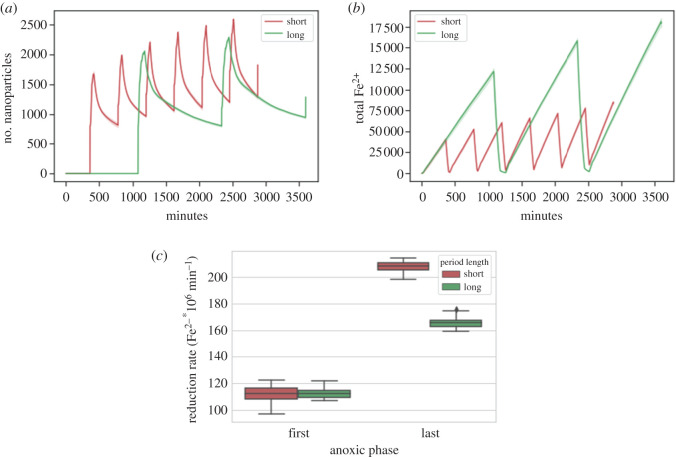
(*a*) Nanoparticle count, (*b*) total Fe^2+^ and (*c*) boxplot of the reduction rates of the first and last anoxic phase for short and long periods of oxic-anoxic cycles. The lag time t was 200 min and the loss rate of reduction susceptibility was 0.003. The plots show the average of 20 runs.

Barcellos *et al.* [68] found that shorter intervals of oxic and anoxic conditions lead to higher Fe^3+^ reduction rates compared to longer intervals if the total incubation time is identical, although the Fe^3+^ reduction rates at the start are the same. Our model is able to reproduce this finding after the inclusion of one simple rule of aged iron minerals being less prone to reduction than fresh iron minerals [69], which could be due to secondary mineral formation. If not interrupted by Fe^3+^ reduction, crystallization processes can lead to the transformation of oxides in minerals which are harder to reduce for the bacteria [70,71]. Thus, we implemented a simplified version of secondary mineral formation: if a nanoparticle has not been reduced for a certain time *t* its reduction susceptibility starts to linearly decrease by a rate *r*. This enables us to reproduce the aforementioned experimental observation qualitatively, as can be seen in figure 2.

The gradual increase of the nanoparticle pool by the FeOB results in a simultaneous increase of the overall Fe^3+^ reduction rate of the system. Although the Fe^3+^ reduction rates between short and long periods of anoxic cycles show no significant difference after the first anoxic phase, the reduction rate for short periods increases twofold compared with the reduction rate for long periods (figure 2*c*). Thus, the pool of nanoparticles increases for both conditions but over long periods the minerals are on average less susceptible to reduction due to higher cristallinity.

Experimental studies focus on isolated aspects of the system, such as reduction rates [29,68,69,72] or the percentage of attached FeRB [31,73,74]. Those studies are used for the parametrization of our model and can therefore not be re-used for validation. The aim of our model is not a quantitatively accurate prediction for specific environmental conditions but a framework for testing general hypotheses about qualitative system behaviour. Very few studies focus on these patterns at the moment. Rather they tend to focus on single species in well-adjusted laboratory environments dealing with a single system variable. Therefore, it has been hard to find a suitable validation for our model. Nevertheless, we carefully collected information from the aforementioned studies pursuing a reductionist approach to implement and parametrize the key processes in the interaction of iron cycling bacteria. As our model provides testable hypotheses, for example, about the relevance of nanoparticles for total system reduction rates under different pH conditions, we deem it likely to provide the first step towards a systemic understanding of this intricately complex system.

### 3.2. Influence of different modes of action

**Figure 3.**
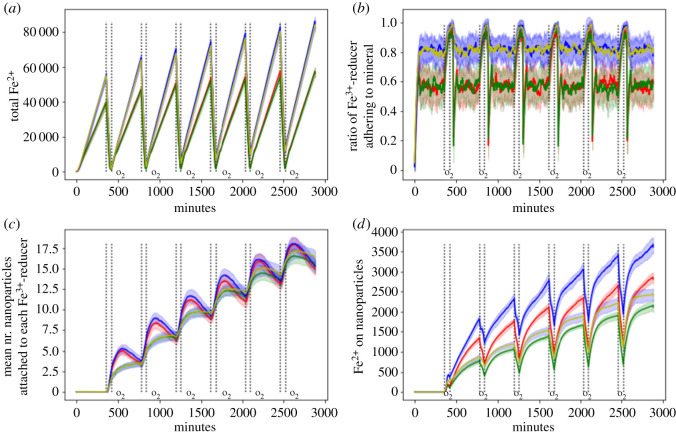
(*a*) Total Fe^2+^, (*c*) ratio of FeRB adhering to mineral, (*c*) mean number of nanoparticles attached to each FeRB and (*d*) Fe^2+^ on nanoparticles for the different combinations of modes of action. Comb. 1 (red): electrostatic adhesion and shedding, Comb. 2 (green): electrostatic adhesion and low pH, Comb. 3 (blue): polymeric adhesion and shedding, Comb. 4 (yellow): polymeric adhesion and low pH. The area ± 1 s.d. (20 replicates per experimental condition) was shaded in a fainter version of the original line colour.

The adhesion mode of *Shewanella* spp. has a small influence on the cycling of iron, whereas the encrustation prevention mode of *Sideroxydans* spp. barely makes a difference in terms of total iron reduction/oxidation (figure 3*a*). This is due to the increased adhesion of *Shewanella* spp. if mediated by polymers (figure 3*b*). As opposed to the electrostatic adhesion, polymer-based adhesion does not allow for the immediate escape from the bulk macroaggregate surface on changing conditions. This means that on average more FeRB stay adhered to the bulk macroaggregate and are able to constantly reduce bulk macroaggregate iron.

As the key factor causing detachment is the excess of Fe^2+^ on the surface, which in turn is strongly dependent on the presence or the absence of oxygen, the adhesion pattern shows strong oscillations under electrostatic adhesion. This oscillatory pattern is dampened under polymeric adhesion because of the delay in detachment. This has the disadvantage that when oxygen subsides and the nanoparticle number is highest, *Shewanella* spp. is not able to take advantage of it by quickly detaching from the bulk macroaggregate and capturing the free-floating nanoparticles which can be reduced more efficiently. Under the conditions tested, which assume an equal number of FeOB and FeRB, our results suggest it is more advantageous to stay adhered to the bulk macroaggregate and constantly reduce the bulk macroaggregate iron, since nanoparticle Fe^3+^ is only a minor fraction of total Fe^3+^. However, this could change with a growing number of FeOB as production of nanoparticles increases so that electrostatic adhesion might surpass polymeric adhesion as a strategy in terms of the amount of iron reduced.

### 3.3. Influence of changing pH-values

**Figure 4.**
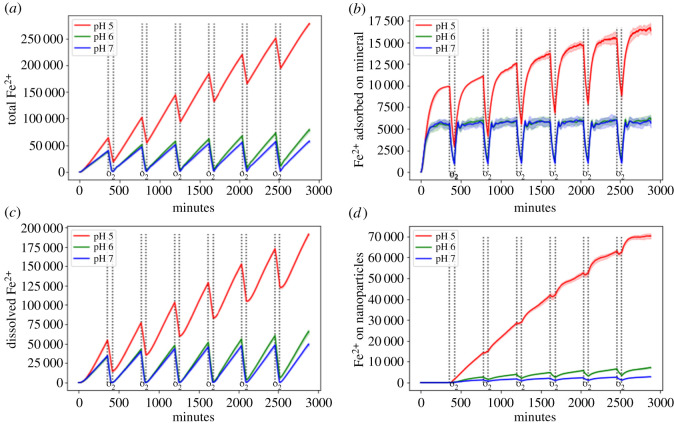
Influence of pH on the concentration of (*a*) total Fe^2+^, (*b*) Fe^2+^ attached to minerals, (*c*) dissolved Fe^2+^ and (*d*) Fe^2+^ on nanoparticles. The shorter and larger intervals between two dotted lines correspond to oxic and anoxic conditions, respectively. The area ±1 s.d. (20 replicates per experimental condition) was shaded in a fainter version of the original line colour.

**Figure 5.**
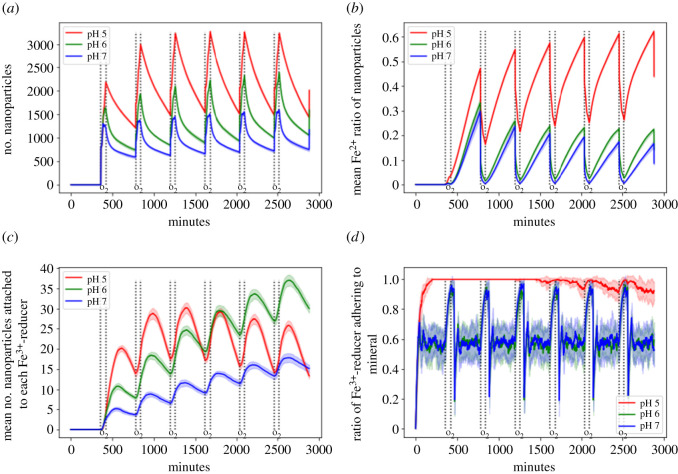
Influence of pH on (*a*) number of nanoparticles, (*b*) mean Fe^2+^ divided by the total Fe-content of each nanoparticle, (*c*) mean number of nanoparticles attached to the FeRB and (*d*) ratio of FeRB adhering to mineral. The shorter interval between two dotted lines encloses oxic conditions, the larger interval anoxic conditions. The area ±1 s.d. (20 replicates per experimental condition) was shaded in a fainter version of the original line colour.

The production of Fe^2+^ is most prevalent under low pH (figure 4). This seems to be mainly due to the stronger tendency of *Shewanella* spp. to adhere to mineral surfaces and therefore being able to constantly reduce Fe^3+^ (figure 5*d*). Although under low pH FeRB show a fast initial increase of nanoparticles attached to their cell surface, the number then steadily declines (figure 5*a*,*c*). The fact that the FeRB spent less time swimming in the medium due to stronger adhesion to the mineral partially explains this finding. The other reason is the high Fe^2+^-concentration in the medium, which causes the nanoparticles to have a high Fe^2+^ to Fe^3+^ ratio (figure 5*b*), thus preventing their binding to the FeRB cell surface due to electrostatic repulsion (figure 5*c*). Therefore, their benefit for microbial reduction consists of providing an additional sink for the adsorption of Fe^2+^ to slow down the accumulation of Fe^2+^ on the surfaces (figure 4*d*), allowing the FeRB to adhere longer to the iron macroaggregate surface and constantly reduce Fe^3+^.

Our results show that the binding of nanoparticles is critically dependent on the pH with optimal binding at pH 6. At a lower pH, the direct interaction between FeRB and the nanoparticles might decline and nanoparticles primarily influence the system as a sink for the reaction product. However, most FeRB are neutrophilic and are not active below pH 6 or 5. Laboratory experiments are needed to determine the optimal pH for an efficient binding and transformation of the nanoparticles.

### 3.4. Sensitivity analysis

**Figure 6.**
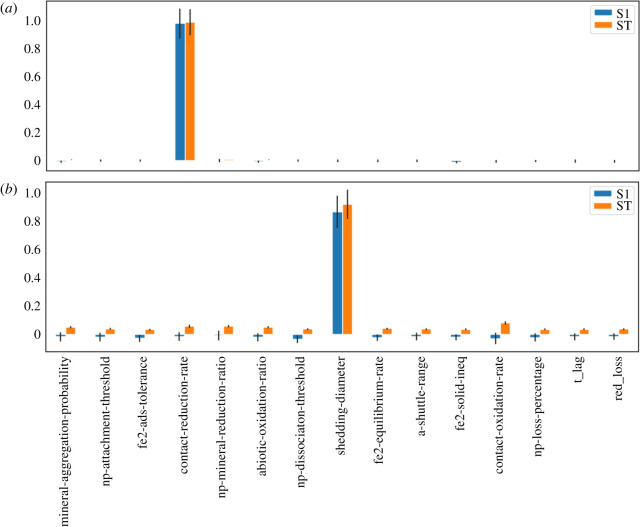
First-order (S1) and total (ST) Sobol indices for all the parameters included in the sensitivity analysis on (*a*) the reduction rate of the last anoxic phase, and (*b*) the number of nanoparticles at the end of the simulation.

Sensitivity analysis provides a method for testing the influence of the parameter values on the measured output variables of the model. Because the intention of our model is to provide a framework for qualitative hypothesis generation and verification, multiple options are conceivable to be picked as the output variables. For this study, we chose the Fe^3+^ reduction rate during the last anoxic phase and the number of nanoparticles at the end of the simulation. Both are of importance for bioremediation applications since they represent an approximation for the steady-state remediation rate. Figure 6 gives an overview of the first-order and total Sobol indices for the output variables tested.

The first-order Sobol indices quantify the influence of varying the respective parameter on the output variable without consideration of interactions with other parameters. Total Sobol indices measure the influence of parameter variations on the output variables with consideration of all possible interactions with other parameters [67]. For the Fe^3+^ reduction rate the situation turned out to be simple: the contact-reduction rate of the FeRB has a dominating influence on the reduction rate.

In strong contrast, the number of nanoparticles is heavily influenced by many parameters. There is still a dominating parameter, the shedding-diameter (the diameter of nanoparticles around which they are released upon growth on the FeOBs cell surface), but all parameters influence the output and do so by complex interactions with each other as the first-order Sobol indices are most of the time negligible compared to the total Sobol indices. The shedding-diameter is so influential as a higher value indicates that the nanoparticles are detached later from the FeOB and therefore smaller nanoparticles are released into the environment.

**Figure 7.**
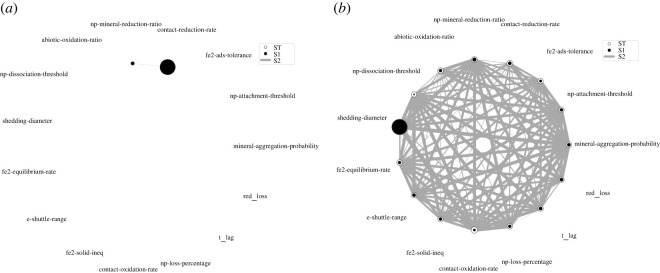
Interaction network of second-order (S2) Sobol indices for the sensitivity analysis on (*a*) the Fe^3+^ reduction rate of the last anoxic phase, and (*b*) the number of nanoparticles at the end of the simulation. The ratio between the inner and outer circle areas clarifies the contribution of the first-order Sobol index (S1) to the total index (ST). The line thickness of the interaction network is a measure for the second-order Sobol index.

More interesting are the less obvious interaction patterns which influence the model output. To have a closer look on the interactions between the parameters, we visualized the network of second-order interactions (figure 7). For the Fe^3+^ reduction rate, only a small second-order interaction exists between the contact-reduction-rate and the np-mineral-reduction-ratio. The first parameter is a measure of how fast Fe^3+^ is reduced in a contact-dependent manner, whereas the np-mineral-reduction-ratio indicates how much faster the FeRB reduces nanoparticles compared to bulk macroaggregate. If both parameter values increase, there is a synergistic effect on the FeRBs’ nanoparticle reduction rate. Again, the sensitivity analysis on the nanoparticle number shows a much more complex interaction network. The nanoparticle number is strongly susceptible to interactions between the different parameters. Interestingly, parameters such as np-dissociation-threshold exhibit their influence primarily through interactions, whereas others, for example the shedding-diameter, affect the nanoparticle number regardless of interactions with other parameters. This is important for the genetic optimization of bacteria for bioremediation purposes as focusing on parameters which have a direct influence on the target variable (high S1/ST ratio) has more predictable outcomes upon manipulation.

## 4. Conclusion

This work proposes the first ABM depicting Fe^3+^ reduction and Fe^2+^ oxidation mediated by two model bacteria. The pH value of the environment is of crucial importance, as efficient reduction of iron nanoparticles takes place at a pH around 6. If the pH exceeds 6, then the high aggregation of nanoparticles leads to a declining number of nanoparticles. Contrarily, if the pH falls below 6, the high Fe^2+^ concentration due to mineral reduction causes the nanoparticles to passivate. Electrostatic repulsion prevents adhesion to the cell surface of the FeRB under these conditions. Our findings are of particular importance for proposed bioremediation attempts which suggest stimulating the oxidation of organic contaminants by FeRB via the injection of nano-sized iron oxides [75]. This strategy could be limited to habitats with a narrow pH range, since the prerequisite for this strategy to work is efficient binding and reduction of nanoparticles.

Our model provides a framework to easily implement and test the qualitative influence of different modes of action on the iron cycling system. For example, the exact mechanism through which the FeOB *Sideroxydans* spp. prevents cell encrustation by iron oxides is not well characterized. Testing the different modes of action in our model provides hints as to which of them might make a difference and which have a negligible influence on the outcomes. According to our results, the encrustation prevention mode of the FeOB has little influence on the total rate of iron reduction, but does have an effect on how big the contribution of nanoparticles to the overall reduction rate is. The nanoparticle reduction is higher if the nanoparticles are directly released into the environment and do not first precipitate on the cell surface.

Through sensitivity analysis for any desired output variable, the model also enables a screening of influential processes and interactions for certain system characteristics. As an example, we showed that the Fe^3+^ reduction rate is mainly influenced by only one parameter, whereas the number of nanoparticles is determined by multiple parameters with parameter interactions playing a major role. The model also enables us to test hypotheses for the explanation of experimental phenomena. For example, we confirmed increasing reduction rates with shorter periods of oxic–anoxic cycles can be attributed to the build-up of nanoparticles and the loss in Fe^3+^ reduction susceptibility due to secondary mineral formation [68].

As described in the Methods section, the parameter values used in this study are based on experimental data and existing literature. At times this was not possible due to the lack of experimental evidence, the variety of strategies or the complexity of the underlying processes. These may be perceived as the inevitable downsides of doing pioneering work in modelling a complex system, but, in a dialectic process the model aims to overcome any shortcomings. Our model provides a flexible framework to test which aspects of the system are most important for any particular output variable of interest. As NetLogo is an accessible programming language, the model can readily be extended with processes of varying complexity. This allows for testing different hypotheses for the explanation of experimental phenomena and will prove helpful in situations where experiments are inaccessible or difficult to carry out.

## Data Availability

Data and relevant code for this research work are stored in GitHub: https://github.com/Athen-Projects/Iron-Cycling and have been archived within the Zenodo repository: https://doi.org/10.5281/zenodo.6482962. The code for conducting the data analysis is provided in the electronic supplementary material [76].
